# A Review of Angular Leaf Spot Resistance in Common Bean

**DOI:** 10.2135/cropsci2018.09.0596

**Published:** 2019-06-04

**Authors:** Michelle M. Nay, Thiago L. P. O. Souza, Bodo Raatz, Clare M. Mukankusi, Maria C. Gonçalves-Vidigal, Angela F. B. Abreu, Leonardo C. Melo, Marcial A. Pastor-Corrales

**Affiliations:** 1 ETH Zürich, Molecular Plant Breeding, Zürich, Switzerland; 2 EMBRAPA Arroz e Feijão, Santo Antônio de Goiás, Goiás, Brazil; 3 Centro Internacional de Agricultura Tropical (CIAT), Cali, Colombia; 4 CIAT, Kawanda, Uganda; 5 Dep. Agronomia, Univ. Estadual de Maringá, Maringá, Paraná, Brazil; 6 Soybean Genomics and Improvement Lab., USDA-ARS, Beltsville, MD 20705

## Abstract

Angular leaf spot (ALS), caused by *Pseudocercospora griseola*, is one of the most devastating diseases of common bean (*Phaseolus vulgaris* L.) in tropical and subtropical production areas. Breeding for ALS resistance is difficult due to the extensive virulence diversity of *P. griseola* and the recurrent appearance of new virulent races. Five major loci, *Phg-1* to *Phg-*5, conferring ALS resistance have been named, and markers tightly linked to these loci have been reported. Quantitative trait loci (QTLs) have also been described, but the validation of some QTLs is still pending. The *Phg-1*, *Phg-4*, and *Phg-5* loci are from common bean cultivars of the Andean gene pool, whereas *Phg-2* and *Phg-3* are from beans of the Mesoamerican gene pool. The reference genome of common bean and high-throughput sequencing technologies are enabling the development of molecular markers closely linked to the *Phg* loci, more accurate mapping of the resistance loci, and the comparison of their genomic positions. The objective of this report is to provide a comprehensive review of ALS resistance in common bean. Furthermore, we are reporting three case studies of ALS resistance breeding in Latin America and Africa. This review will serve as a reference for future resistance mapping studies and as a guide for the selection of resistance loci in breeding programs aiming to develop common bean cultivars with durable ALS resistance.

Common bean (*Phaseolus vulgaris* L.), which includes dry and snap beans, is the world’s most important grain legume for direct human consumption and is also an important source of protein, fiber, calories, and vital micronutrients, particularly for millions of people in Latin America and eastern and southern Africa (Singh, 1999; Broughton et al., 2003). Frequent consumption of dry seeds of common bean combined with cereals ensures a balanced diet of essential amino acids and other nutrients that contribute to alleviating malnutrition and preventing cardiovascular disease, diabetes, and certain types of cancer (Broughton et al., 2003; Thompson et al., 2017; Viguiliouk et al., 2017).

The Americas are the largest common bean-producing region, and Brazil is the world’s largest producer and consumer (Singh, 1999; Beebe, 2012). Africa, where common bean was introduced after the discovery of the Americas, is second in production. Moreover, consumption in several African countries, up to 66 kg person^−1^ yr^−1^, is greater than that in Latin America (Wortmann et al., 1998; Singh, 1999; Broughton et al., 2003; Beebe, 2012).

Numerous infectious diseases caused by fungi, viruses, bacteria, and nematodes represent major limitations to common bean production throughout the world (Schwartz and Pastor-Corrales, 1989; Schwartz et al., 2005). Angular leaf spot (ALS), a disease caused by *Pseudocercospora griseola* (Sacc.) Crous & Braun [previously referred to as *Phaeoisariopsis griseola* (Sacc.) Ferrari], was until the 1980s considered to be of minor importance in Latin America, particularly in Brazil (Rava Seijas et al., 1985). However, in the mid-1980s, ALS began to be considered a significant constraint to dry bean production in Brazil, Central America, and eastern and southern Africa (Rava Seijas et al., 1985; Liebenberg and Pretorius, 1997; Pastor-Corrales et al., 1998; Aggarwal et al., 2004). Angular leaf spot is currently one of the most recurring and devastating diseases of dry beans in Latin America and Africa, the most important production areas of the world (Correa-Victoria. et al., 1989; Liebenberg and Pretorius, 1997; Wortmann et al., 1998; Stenglein et al., 2003; Sartorato, 2004; Crous et al., 2006). Angular leaf spot has also been reported to occur sporadically in countries of the temperate climate zone, including the United States and Canada (Correa and Saettler, 1987; Melzer and Boland, 2001), and ALS was recently reported for the first time in northern Spain (Landeras et al., 2017).

Yield losses caused by ALS can reach up to 80% (Schwartz et al., 1981; Rava Seijas et al., 1985; de Jesus Junior et al., 2001). Although fungicides are an option for the control of ALS, they are often expensive or not readily available to smallholder farmers, the predominant producers of dry beans in the tropics. Cultivars with resistance to *P. griseola* offer a cost-effective, easy-to-use, and environmentally friendly management strategy (Pastor-Corrales et al., 1998), and several sources of ALS resistance have been identified among primary and secondary gene pools of *P. vulgaris* (CIAT, 1996, 1999; Pastor-Corrales et al., 1998; Busogoro et al., 1999a; Mahuku et al., 2003, 2009, 2011). However, development of common bean cultivars with durable ALS resistance is difficult due to the broad and changing virulence diversity of the ALS pathogen that renders varieties that are resistant in one year or location susceptible in another (Pastor-Corrales et al., 1998; Mahuku et al., 2002b).

Resistance to the ALS pathogen is largely conferred by single dominant resistance genes, hereafter also referred to as loci. Recent studies also show a more quantitative nature of resistance that includes quantitative trait loci (QTLs). To date, five ALS resistance loci have been approved by the Bean Improvement Cooperative (BIC) Genetics Committee (http://arsftfbean.uprm.edu/bic/wp-content/ uploads/2018/04/Bean_Genes_List_2017.pdf) that maintains the guidelines for the nomenclature of disease resistance genes in common bean. These include three dominant and independent *Phg* loci named *Phg-1*, *Phg-2*, and *Phg-3* and two major QTLs named *Phg-4* and *Phg-5* (de Carvalho et al., 1998; Sartorato et al., 1999a; Corrêa et al., 2001; Namayanja et al., 2006; Gonçalves-Vidigal et al., 2011, 2013; Oblessuc et al., 2012, 2013; Keller et al., 2015).

New resources, including the reference genome of common bean (Schmutz et al., 2014) and high-throughput sequencing, facilitate the development of different types of molecular markers that are tightly linked to these loci and provide new insight into the relationship between existing and newly discovered disease resistance loci. This review aims to (i) discuss the virulence diversity of *P. griseola* and its impact on disease resistance breeding, (ii) discuss the current knowledge of ALS resistance, (iii) comment on how new genomic resources might facilitate and accelerate ALS research, including gene discovery and the development of highly accurate molecular markers, and (iv) present three case studies of ALS resistance breeding in Brazil, Colombia, and Uganda.

## *PSEUDOCERCOSPORA GRISEOLA*, THE CAUSAL AGENT OF ANGULAR LEAF SPOT DISEASE

The ALS pathogen belongs to the class Dothideomycetes, the largest and most diverse class of ascomycete fungi, which contains many important plant pathogens, endophytes, and saprobes (Crous et al., 2006; Schoch et al., 2009). Although *P. griseola* can be transmitted through seeds, the most frequent source of primary inoculum to initiate ALS disease under natural conditions is the presence of plant debris infected with the pathogen (Correa-Victoria. et al., 1989). *Pseudocercospora griseola* is considered a fastidious pathogen (Correa-Victoria et al., 1989), yet it grows and produces spores on artificial culture media. Lyophilization has been successfully used for the long-term storage of spores (Castellanos et al., 2016). The response of common bean germplasm to *P. griseola* is usually evaluated using a disease severity scale ranging from 1 to 9, where scores of 1 to 3 are considered resistant, 4 to 6 are intermediate, and 6 to 9 are susceptible (van Schoonhoven and Pastor-Corrales, 1987).

The ALS pathogen is known for its extensive virulence diversity (Guzman et al., 1995; Chacón et al., 1997; Pastor-Corrales et al., 1998; Busogoro et al., 1999b; Mahuku et al., 2002b; Aggarwal et al., 2004; Sartorato, 2004). In the early 1980s, while working in the Bean Program of CIAT, Cali, Colombia, the corresponding author of this manuscript developed a set of 12 common bean differential cultivars: six Andean and six Mesoamerican. To standardize races of *P. griseola* (Table 1), a binary code was implemented and launched in 1995 during the first ALS workshop (CIAT, 1995).

**Table 1:** Set of 12 common bean differential cultivars and binary code used to designate races of *Pseudocercospora griseola*. Each race is assigned two numbers based on the summation of the binary numbers of the susceptible Andean and Mesoamerican cultivars, respectively. The example in the table shows how a Mesoamerican isolate of *P. griseola* was characterized as race 25-39.

Differential cultivars	Seed size	Common bean race	Resistance gene/ linkage group	Binary value	Reaction and binary value of susceptible cultivars
Andean cultivars†					
Don Timoteo	Large	Chile	Unknown	1	Susceptible: 1
G11796	Large	Peru	Unknown	2	Resistant
Bolon Bayo	Large	Peru	Unknown	4	Resistant
Montcalm	Large	Nueva Granada	Unknown	8	Susceptible: 8
Amendoim	Large	Nueva Granada	Unknown	16	Susceptible: 16
G5686	Large	Nueva Granada	*Phg-4*/Pv04; *Phg-5*/Pv10	32	Resistant
Mesoamerican cultivars‡					
PAN 72	Small	Mesoamerica	Unknown	1	Susceptible: 1
G2858	Medium	Durango	Unknown	2	Susceptible: 2
Flor De Mayo	Small	Jalisco	Unknown	4	Susceptible: 4
Mexico 54	Medium	Jalisco	*Phg-2*/Pv08	8	Resistant
BAT 332	Small	Mesoamerica	*Phg-2^2^*/Pv08	16	Resistant
Cornell 49-242	Small	Mesoamerica		32	Susceptible: 32

† Andean binary value (1 + 8 + 16) = 25.

‡ Mesoamerican binary value (1 + 2 + 4 + 32) = 39 (race 25–39).

Characterization of the virulence phenotype (known as races) of isolates of *P. griseola* on Andean and Mesoamerican common bean differential cultivars has resulted in the separation of these isolates into two distinct virulence groups (Pastor-Corrales and Jara, 1995). Isolates obtained from large-seeded bean cultivars of the Andean gene pool from Ecuador, Colombia, and Argentina were virulent only on Andean differential bean cultivars, and these races were referred to as Andean. Isolates from small- and medium-seeded Mesoamerican differential cultivars from Central America, Brazil, Bolivia, and Argentina were virulent on both Mesoamerican and Andean ALS differential cultivars and were referred to as Mesoamerican (Beebe and Pastor-Corrales, 1991; Guzman et al., 1995; Pastor-Corrales and Jara, 1995; Pastor-Corrales et al., 1998; Mahuku et al., 2002b; Stenglein and Balatti, 2006).

Similar studies using differential cultivars and molecular techniques have revealed that the virulence and genetic diversity of two other pathogens that cause anthracnose (*Colletotrichum lindemuthianum*) and rust (*Uromyces appendiculatus*) also segregate into two distinct groups that mirror the diversity of their common bean host (Beebe and Pastor-Corrales, 1991; Pastor-Corrales, 1996; Pastor-Corrales and Aime, 2004).

The set of differential cultivars has been extensively used throughout the world and has permitted the comparison of races of the ALS pathogen between continents, countries, and locations. In general, races of *P. griseola* differ between regions, countries, and even continents.

Some of the most aggressive isolates of *P. griseola*, belonging to race 63-63, that are virulent on all Andean and Mesoamerican differential cultivars were first observed in Latin America. These races have been recurrently found in Brazil, Argentina, and Central America (Mahuku et al., 2002b; Sartorato, 2004; Stenglein and Balatti, 2006; Silva et al., 2008). Later, these races were infrequently found in Africa (Mahuku et al., 2002a; Wagara et al., 2004, 2011). Although Andean and Mesoamerican races have been found in the Americas and Africa, their predominance on these continents differ. Initially, Andean races infecting only Andean differential cultivars were the predominant races in Africa (Guzman et al., 1995; Aggarwal et al., 2004). Shortly afterward, the first reports of races that infected mainly Andean differentials but also a few Mesoamerican differential cultivars appeared. These races were termed Afro-Andean (CIAT, 1997; Mahuku et al., 2002a; Wagara et al., 2004, 2011). Using molecular markers to analyze the diversity of Afro-Andean races, Mahuku et al. (2002b) suggested that the Afro-Andean races belonged to the Andean group, disputing the existence of the Afro-Andean group.

Recently, Serrato-Diaz et al. (personal communication, 2018) further investigated the population structure of *P. griseola* by sequencing four nuclear genes to construct a phylogenetic tree. These authors found that isolates from Puerto Rico, Honduras, and Guatemala clustered in the Mesoamerican clade, whereas the Tanzanian isolates clustered into three clades: Mesoamerican, Andean, and Afro-Andean. These findings suggest a more diverse population structure than the previously reported Mesoamerican and Andean groups, but additional research is needed to resolve the extent of the population structure of this pathogen.

Although differential cultivars provide a simple and effective method for studying pathogen virulence diversity, some or all differential cultivars may become susceptible to new virulent isolates due to changes in the virulence spectra of pathogens over time and space. In fact, the virulence diversity of *P. griseola* appears to have changed. Many new isolates belonging to race 63-63 that are virulent on all differential cultivars have been found in Latin America and Africa (Sartorato, 2004; Wagara et al., 2004, 2011; Stenglein and Balatti, 2006; Silva et al., 2008; Ddamulira et al., 2014b; Pereira et al., 2015). Because of the increased occurrence of isolates of race 63-63, efforts are underway to develop a new set of differential cultivars, and in 2015, new candidates were proposed during the Common Bean Disease Workshop in Skukuza, South Africa. However, before a new set of differential cultivars can become available, they need to be tested for their reaction to *P. griseola* isolates from different countries and, in particular, for reactions to isolates of race 63-63.

Because of the high virulence diversity of *P. griseola* and the great potential for overcoming resistance, a successful resistance breeding strategy requires a sound understanding of the virulence diversity and evolution of this pathogen. Based on what is known about the parallel evolution between gene pools of the common bean host and pathogen, proposed strategies for durable ALS resistance include pyramiding of Andean and Mesoamerican resistance genes or using Mesoamerican resistance sources in areas where predominantly Andean isolates exist, and vice versa (Guzman et al., 1995; Pastor-Corrales et al., 1998).

## OVERVIEW OF MAJOR LOCI CONDITIONING RESISTANCE TO *PSEUDOCERCOSPORA GRISEOLA*

Since the beginning of the 1980s, many studies have reported new sources and genes conferring resistance to ALS, as well as molecular markers linked to ALS resistance genes (Schwartz et al., 1982). However, many of these publications did not include appropriate physical linkage information or allelism tests and thus could not be validated. Only well-characterized loci or QTLs for which linked molecular markers are available can be submitted for acceptance to the BIC Genetics Committee. The currently approved ALS resistance loci include three dominant and independent loci, *Phg-1*, *Phg-2*, and *Phg-3*, as well as two major QTLs, *Phg-4* and *Phg-5* (Table 2).

**Table 2:** Named and mapped angular leaf spot resistance genes in common bean. Resistance loci are stated with their new name accepted by the Bean Improvement Cooperative Genetics Committee and their originally published name. Table modified from Souza et al. (2016).

Locus symbol		Resistance source	Gene pool†	Chromosome	Pathogen race	Reference
New	Original					
Phg-1	Phg-1	AND 277	A	Pv01	63-23	de Carvalho et al. (1998), Gonçalves-Vidigal et al. (2011)
Phg-2	Phg-2	Mexico 54	MA	Pv08	63-19	Sartorato et al. (1999a), Sartorato et al. (2000)
Phg-2^2^	–	BAT 332	MA	Pv08	63-39	Namayanja et al. (2006)
Phg-3	Phg-ON	Ouro Negro	MA	Pv04	63-39	Corrêa et al. (2001), Gonçalves-Vidigal et al. (2013)
Phg-4	PhgG_5686A_, ALS4.1^GS,UC^	G5686	A	Pv04	31-0, 31-0	Mahuku et al. (2009), Keller et al. (2015)
Phg-5	ALS10.1^DG,UC^	CAL 143	A	Pv10	0-39, field	Oblessuc et al. (2012, 2013)
Phg-5	ALS10.1^DG,UC,GS^	G5686	A	Pv10	31-0	Keller et al. (2015)

† A, Andean; MA, Mesoamerican.

**Fig.1:**
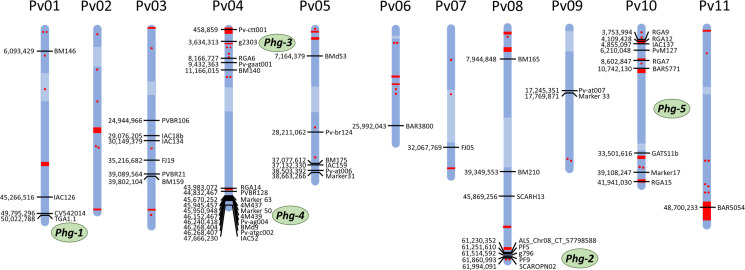
Genetic map showing positions of reported markers tagging angular leaf spot (ALS) resistance loci. Markers linked to ALS resistance loci are shown with their location mapped on the Phaseolus vulgaris reference genome version 2.1 (available at https://phytozome.jgi.doe.gov/pz/portal.html). The resistance genes approved by the Bean Improvement Cooperative Genetics Committee and their approximate positions are marked in green on the right side of the chromosome. Centromere regions are shown in light blue as reported in the reference genome (Schmutz et al., 2014). Resistance genes, containing an ARC domain (PF00931) are marked in red, with points if there are less than three loci and with bands if there are three or more loci. A summary of markers linked to resistance loci and their primer sequences is given in the Supplemental Table S1.

Methods of characterizing ALS resistance loci have changed over time. Initial work with random amplification of polymorphic DNA (RAPD), amplified fragment length polymorphism (AFLP), and restriction fragment length polymorphism (RFLP) markers was followed by sequence characterized amplified region (SCAR), simple sequence repeat (SSR), and single-nucleotide polymorphism (SNP) marker systems (Semagn et al., 2006; Jiang, 2013; Fang, 2015). The publication of the common bean reference genome (Schmutz et al., 2014) has permitted the mapping and comparison of the positions of most SCAR, SSR, and SNP markers (Fig. 1). In this section, we discuss the progress in ALS resistance characterization, focusing mainly on genetic mapping studies and remapping molecular markers linked to ALS resistance genes on the reference genome of common bean. For details on markers, see Supplemental Table S1.

### Phg-1

#### Origin

The *Phg-1* locus was reported on chromosome Pv01, and it is tightly linked to the anthracnose resistance locus *Co-1^4^* in the Andean cultivar AND 277 (Gonçalves-Vidigal et al., 2011).

#### Molecular Markers

The *Phg-1* and *Co-1*4 loci are tightly linked (0.0 cM) to each other on chromosome Pv01 (Gonçalves-Vidigal et al., 2011). Two molecular markers, CV542014^450^ and TGA1.1^570^, flanking the *Co-1^4^/Phg-1* loci were identified as linked at 0.7 and 1.3 cM, respectively.

#### Alleles

No alleles were reported.

#### Breeding Value

The cultivar AND 277, which was obtained from the cross of Andean cultivars G 21720 ´ BAT 1386, is an important ALS resistance source that has been used in breeding programs in Brazil and southern Africa (de Carvalho et al., 1998; Aggarwal et al., 2004; Arruda et al., 2008). AND 277 was reported to be resistant to *P. griseola* and *C. lindemuthianum* under field conditions during 2 yr of evaluations in Malawi (Aggarwal et al., 2004). The ALS-resistant Andean cultivar CAL143, derived from a cross G12229 ´ AND 277, may carry *Phg-1* present in the AND 277 parent; however, there are no studies supporting this assertion (Aggarwal et al., 2004). CAL 143 is a high-yielding variety that has a strong level of resistance to ALS, rust, and halo blight (caused by *Pseudomonas syringae* pv. *phaseolicola*) under field and greenhouse conditions (Chataika et al., 2010; Oblessuc et al., 2012; Cichy et al., 2015).

### Phg-2

#### Origin

The *Phg-2* locus was discovered in Mesoamerican cultivar Mexico 54 as a single dominant resistance locus on chromosome Pv 08 (Sartorato et al., 1999a).

#### Molecular Markers

Sartorato et al. (1999a) reported that the RAPD markers OPN 02, OPN 14, and OPE 04 were linked to *Phg-2* in Mexico 54 at 5.9, 6.6, and 11.8 cM, respectively. The SCAR marker N02, which was developed based on the SN02_890_ fragment, showed polymorphisms identical to the original RAPD OPN 02 marker (Sartorato et al., 1999b, Sartorato et al., 2000). However, the N02 marker was not polymorphic in other evaluated populations using Mexico 54 as the resistant parent (Namayanja et al., 2006). The polymerase chain reaction (PCR) marker g796, which is highly specific for Mexico 54, was linked to the *Phg-2* locus at 3 cM distance (Miller et al., 2018).

#### Alleles

In addition to Mexico 54, other Mesoamerican cultivars including Cornell 49-242, MAR 2, G10474, BAT 332, and G10909 contain ALS resistance loci that map to the lower end of chromosome Pv08. . Physical position analysis using the common bean reference genome sequence (Schmutz et al., 2014) indicated that the ALS resistance in these cultivars may be conferred by alleles of *Phg-2* (Souza et al., 2016). However, this information requires verification.

A single dominant resistance locus was found in cultivar Cornell 49-242 controlling resistance to race 31-17 (Nietsche et al., 2000; Caixeta et al., 2005). The OPN02_890_ and OPE04_650_ RAPD markers linked to *Phg-2* in Mexico 54 were also found to be linked to the resistance locus in Cornell 49-242 at 3.2 and 12.5 cM, respectively (Nietsche et al., 2000). Given that the same markers are linked to ALS resistance in Mexico 54 and Cornell 49-242, these loci might be allelic (Nietsche et al., 2000). OPE04_650_ was also found to be linked at 5.8 cM to a single dominant resistance locus in MAR 2 conferring resistance to race 63-39 (Ferreira et al., 2000).

Two genes in bean genotype G10909, on chromosomes Pv04 and Pv08 confer resistance to the highly virulent race 63-63 (Mahuku et al., 2011). The resistance gene *Phg_G10909B_* on chromosome Pv08 was found to cosegregate with SCAR markers PF13_310_, PF9_260_, and OPE04_709_ at 4.9, 7.4, and 9.9 cM, respectively (Mahuku et al., 2011).

A single and dominant gene in cultivar BAT 332 linked to RAPD markers OPAA07_950_ and OPAO12_950_ at 5.10 and 5.83 cM, respectively, confers resistance to race 61-41 (Caixeta et al., 2003). Additionally, an allelism test between Mexico 54 and BAT 332 inoculated with race 63-39 showed no segregation, which is an indication that the loci conferring ALS resistance in Mexico 54 and BAT 332 are allelic (Namayanja et al., 2006). Angular leaf spot resistance in BAT 332 has been designated as the *Phg-2^2^* allele. At present, *Phg-2^2^* is the only allele of *Phg-2* officially accepted by the BIC Genetics Committee.

A single dominant resistance gene in Mesoamerican common bean accession G10474 was linked to the codominant SCAR marker PF5, positioned 5.0 cM from the resistance locus. The gene-pool-specific PF5 marker can be used to transfer resistance from G10474 to Andean common bean cultivars (Mahuku et al., 2004). In addition, another highly specific marker, ALS_Chr08_ CT_57798588, found through whole-genome sequencing of G10474, can be used in marker-assisted selection (MAS) (Lobaton et al., 2018).

In summary, the known physical positions of linked markers suggest that the ALS resistance genes in MAR 2, Cornell 49-242, G10474, and G10909 are either alleles of *Phg-2* from Mexico 54, or they may represent different loci within a resistance gene cluster. Allelism studies have only confirmed that the resistance loci in BAT 332 and MAR 2 are allelic to *Phg-2* (Caixeta et al., 2005; Namayanja et al., 2006). Further genomic characterization of the *Phg-2* locus is necessary to clarify allelic relationships or the presence of potentially different genes within the locus.

#### Breeding Value

The *Phg-2* locus found in Mexico 54 and its potential alleles reported in various Mesoamerican cultivars confer the broadest known resistance. These alleles are present in several bean cultivars used in ALS resistance breeding. Mexico 54 has been extensively used in breeding and research, and it is very resistant to African isolates of *P. griseola*, though this cultivar was resistant to only 5 of 19 Colombian races used in one study (CIAT, 2005; Chilagane et al., 2013; Ng’ayu-Wanjau, 2013; Ddamulira et al., 2015). The putative *Phg-2* allele of G10474 confers broad resistance to most races of *P. griseola*, and it has been extensively used in breeding at CIAT. G10474 was found to be resistant to most races screened under greenhouse conditions. Only races from Haiti and South Africa caused disease in this cultivar (Mahuku et al., 2004, CIAT, 2005).

### Phg-3

#### Origin

The *Phg-3* ALS resistance locus was first reported in the Mesoamerican common bean cultivar Ouro Negro (Corrêa et al., 2001). A later study reported that *Phg-3* cosegregates with the anthracnose resistance locus *Co-3^4^* (previously named *Co-10*), and that *Phg-3*/*Co-3^4^* alleles were tightly linked to each other (0.0 cM) on chromosome Pv04 (Gonçalves-Vidigal et al., 2013).

#### Molecular Markers

Gonçalves-Vidigal et al. (2013) reported that the *Phg-3*/ *Co-3^4^* resistance alleles were tightly linked to the marker g2303, at 0.0 cM on Pv04, enabling the use of MAS to transfer the ALS and anthracnose resistance cluster to commercial bean cultivars.

#### Alleles

The resistance locus *Phg*_G10909A_ in the Mesoamerican cultivar G10909 is located on chromosome Pv04, 13 cM from microsatellite marker Pv-gaat001 (Mahuku et al., 2011). *Phg*_G10909A_ and *Phg-3* are in the same region, though no allelism tests between these loci have been conducted.

#### Breeding Value

Ouro Negro is a highly productive black-seeded Mesoamerican cultivar with desirable agronomic and cooking characteristics that was selected from CIAT accession G3680, also known as Honduras 35 (Alzate-Marin et al., 2003). The *Phg-3* ALS, *Co-3^4^* anthracnose, and *Ur-14* rust resistance alleles present in Ouro Negro are very important for common bean breeding programs in Brazil (Alzate-Marin et al., 2003; Souza et al., 2011; Gonçalves-Vidigal et al., 2013), conferring resistance to at least 21 *C. lindemuthianum* races and seven *P. griseola* races, including highly virulent race 63-63 (Faleiro et al., 2001, 2003; Gonçalves-Vidigal and Kelly, 2006; Gonçalves-Vidigal et al., 2009, 2013; Ragagnin et al., 2009).

### Phg-4

#### Origin

The *Phg-4* locus, previously named *Phg_G5686A_*, was discovered in the Andean cultivar G5686 inoculated with *P. griseola* race 31-0 (Mahuku et al., 2009). Keller et al. (2015) used a fine-mapping approach to characterize and delimit the QTL on chromosome Pv-04 in G5686 and named it ALS4.1^GS, UC^. Because of the consistent and significant effects of this major locus across different environments and populations (Mahuku et al., 2009; Keller et al., 2015), the BIC Genetics Committee has approved the name *Phg-4* for the ALS4.1 QTL (Souza et al., 2016).

#### Molecular Markers

The *Phg-4* locus was found to be linked, at 0.0 cM, to the microsatellite marker Pv-ag004 from G5686 (Mahuku et al., 2009). Keller et al. (2015) used fine mapping to investigate the *Phg-4* locus in detail, delimiting it to a 418-kb genomic region between markers Marker63 and 4M439. The delimited region includes 36 candidate genes, including 11 serine/ threonine protein kinases arranged in a repetitive array, which are promising candidate genes for ALS resistance. Single nucleotide polymorphism-based markers highly specific to *Phg-4* in G5686 are available on several genotyping platforms (Keller et al., 2015; Lobaton et al., 2018)

#### Alleles

Using a CAL 143 (resistant) ´ IAC-UNA (susceptible) cross, Oblessuc et al. (2012) reported two adjacent QTLs, ALS4.1^GS, UC^ delimited by markers IAC52 and BMd9, and ALS4.2^GS, UC^ delimited by markers PVBR92 and Pv-gaat001. Although these two QTLs were reported as being close to the *Phg-4* locus, they have not been approved by the BIC Genetics Committee as alleles of *Phg-4*.

#### Breeding Value

G5686 has been inoculated with >500 isolates of *P. griseola* from 27 countries and found to be one of the most resistant genotypes. G5686 is currently being used in breeding line development at CIAT.

### Phg-5

#### Origin

The resistance locus *Phg-5* on chromosome Pv10 has been found in two different Andean common bean cultivars: CAL 143 and G5686. The *Phg-5* locus was first reported in a CAL 143 ´ IAC-UNA recombinant inbred line (RIL) population evaluated in the field under natural infection and inoculated under greenhouse conditions with *P. griseola* race 0-39 (Oblessuc et al., 2012). *Phg-5*, previously named QTL ALS10.1, exhibits a strong effect in booth, field, and greenhouse environments. Keller et al. (2015) confirmed the presence of *Phg-5* in common bean accession G5686. Besides a rough positional analysis, there is no evidence that *Phg-5* in CAL 143 and G5686 is the same allele or that they are different genes.

#### Molecular Markers

The closely linked markers GATS11b and IAC137 flank the *Phg-5* locus in CAL 143 (Oblessuc et al., 2012). Although the two markers are closely linked, the physical positions of GATS11b (33.50 Mb) and IAC137 (4.86 Mb) are very far apart; this range coincides with the centromeric region of chromosome Pv10, which shows little recombination (Schmutz et al., 2014). Oblessuc et al. (2013) increased the marker density around the *Phg-*5 locus and identified ATA220 marker, which coincided with the peak LOD score, but ATA220 did not map to the common bean genome reference. Investigation of the transcriptional modulation of the *Phg-5* region revealed an enrichment of genes involved in plant–pathogen interactions, and seven of the 323 genes located in the core region were found to be differentially regulated after infection (Oblessuc et al., 2015). Keller et al. (2015) reported a minor QTL linked to Marker17 on chromosome Pv10 in G5686, which was designated as *Phg-5*.

#### Alleles

López et al. (2003) reported four resistance gene analogs on chromosome Pv10 linked to ALS resistance in a RIL population from the G19833 ´ DOR364 cross. Another minor QTL was found to be associated with marker BAR5771 in AND 277 on chromosome Pv10, conferring resistance to race 1-21 (Bassi et al., 2017).

#### Breeding Value

CAL 143 has been used extensively in breeding, and it is a popular variety that has been released in several African countries, including under the name of Lyambai in Zambia. G5686 is one of the most resistant Andean genotypes known, and it carries the resistance locus *Phg-5*, but it also contains *Phg-4* (Mahuku et al., 2009, Keller et al., 2015).

## OTHER REPORTED ANGULAR LEAF SPOT RESISTANCE LOCI TAGGED BY MARKERS

Besides the well-characterized five ALS resistance loci (*Phg-1* to *Phg-5*), other loci have been reported, but they showed either a weak effect on resistance or there was not sufficient evidence to validate their existence and to assign them a *Phg* symbol.

### Chromosome Pv01

In phenotypic evaluations under field conditions of a Jalo EEP 558 (resistant) ´ Small White (susceptible) cross, Teixeira et al. (2005) found an ALS QTL linked to marker BM146 of Jalo EEP 558, which they reported to be on chromosome Pv05. However, this marker has been mapped to the upper end of chromosome Pv01 on the reference genome, with *Phg-1* positioned at the lower end of the same chromosome (Fig. 1).

### Chromosome Pv03

Using an RIL population from the CAL 143 ´ IAC-UNA cross, Oblessuc et al. (2012) found a minor QTL on chromosome 3 flanked by markers PVBR21 and FJ19. These authors also reported an ALS resistance locus on chromosome Pv02 flanked by markers IAC134 and IAC18b. However, these markers have been mapped to chromosome Pv03 of the reference genome, in proximity to other reported markers (Fig. 1). The resistant allele at this locus is derived from CAL 143.

### Chromosome Pv05

Oblessuc et al. (2012) also discovered two additional QTLs on chromosome Pv05 in the CAL 143 ´ IAC-UNA cross. The QTL ALS5.1^UC^ was flanked by markers BMd53 and FJ05, and QTL ALS5.2^UC^, which exhibited a strong effect under greenhouse conditions, was flanked by markers BM175 and IAC261. By mapping the above mentioned markers onto the reference genome, BMd53 and BM175 were confirmed to be located on chromosome Pv05, however; FJ05 and IAC261 were mapped to chromosomes Pv07 and Pv01, respectively (Fig. 1). In another study using RILs from the AND 277 (resistant) ´ SEA 5 cross (susceptible) inoculated with race 1-21, a minor QTL was found associated with marker IAC159 on chromosome Pv05 (Bassi et al., 2017). Quantitative trait locus mapping in G5686 ´ Sprite revealed a minor QTL in the same genomic region that explained 3.7% of the variance associated with Marker 31 (Keller et al., 2015).

### Chromosome Pv06

In the AND 277 ´ SEA 5 RIL population, another minor QTL associated with marker BAR3800 was found on chromosome 6 (Bassi et al., 2017).

### Chromosome Pv08

An ALS resistance QTL was found on the upper arm of chromosome Pv08, opposite to *Phg-2*. Teixeira et al. (2005) found markers BM210 and BM165 to be linked to ALS resistance in Jalo EEP 558. Marker BM165 was initially reported to be on chromosome Pv05, though it maps to chromosome 8 (Fig. 1).

### Chromosome Pv09

A study of G5686 (resistant) ´ Sprite (susceptible) cross revealed a locus named *Phg_G5686C_* on chromosome Pv09 linked, at 12.1 cM, to marker Pv-at007 (Mahuku et al., 2009). This locus was confirmed as a minor QTL explaining 1.7% of the variance linked to Marker 33 (Keller et al., 2015).

### Chromosome Pv11

Bassi et al. (2017) reported a major QTL conferring ALS resistance on chromosome Pv-11, explaining 26.5% of the observed phenotypic variance using RIL of AND 277 ´ SEA 5. Marker BAR5054 was associated with ALS resistance in AND 277, and also with susceptibility to powdery mildew (caused by *Erysiphe poligoni* DC) in AND 277 (Bassi et al., 2017).

## SEGREGATION AND ALLELISM STUDIES

In addition to the abovementioned studies reporting molecular markers linked to ALS resistance loci, numerous other segregation studies have been conducted. In these studies, resistance sources were crossed to susceptible varieties or to other resistance sources, and segregation ratios were analyzed to draw conclusions about the inheritance or allelism of resistance loci (Caixeta et al., 2005; CIAT, 2006; Namayanja et al., 2006; Mahuku et al., 2011). However, these studies were often deficient and did not always support their conclusions. First, ALS resistance is often quantitative; thus, the classification of ALS responses into resistant and susceptible categories is not suitable because the score distribution of resistant and susceptible plants does not segregate into two well-defined groups (Teixeira et al., 2005; Chataika et al., 2010; Oblessuc et al., 2012). Second, when evaluations are conducted using single F_2_ plants, errors can be introduced during phenotyping and due to hybridization problems. Finally, distinct interactions between the resistance loci in common bean and isolates of *P. griseola* exist; thus, the type of resistance loci observed are contingent on the isolate of *P. griseola* that is used. Taken together, the diversity of isolates of the ALS pathogen and technical and statistical issues of most published allelism studies render these results difficult to interpret, thus limiting the knowledge gained from such studies.

## OUTLOOK ON GENETIC CHARACTERIZATION OF ANGULAR LEAF SPOT RESISTANCE

Methods to genetically characterize ALS resistance in common bean have changed substantially in recent years. For instance, the publication of the reference genome of common bean has allowed for the assignment of positions for most SCAR, SSR, and SNP markers and the comparison of loci obtained from different studies. For several genotypes, resistance loci have been mapped to similar positions on the genome. Although mapping studies are often complemented by allelism and segregation analyses, some allelism studies are difficult to interpret and frequently report more and different genes involved in resistance. Overall, additional studies are needed to resolve these discrepancies.

The identification of resistance genes will be a major goal for geneticists in order to understand the nature of defense genes and to define haplotypes for marker design to aid in breeding. In this respect, the locus best characterized to date is *Phg-5* where the expression of its candidate genes has been investigated (Oblessuc et al., 2015). However, fine mapping of *Phg-5* has been hindered by the partial localization of the core QTL region to a pericentromeric region that spans several megabases and where little recombination occurs. In contrast, the *Phg-4* locus has been mapped to a much smaller region in which 36 candidate genes have been annotated (Keller et al., 2015). Although the whole genome sequencing data of G5686 is available, the repetitive nature of the *Phg-4* region poses a barrier to de novo assembly and to the correct identification of all copies of potential resistance genes and polymorphisms. Other technologies, such as 10´ sequencing (www.10xgenomics.com), PacBio (www.pacb.com), and Oxford Nanopore (www.nanoporetech.com), are promising for overcoming the issues of short read assemblies and will allow researchers to assemble repetitive genomic regions more accurately.

Next-generation sequencing technologies have reduced sequencing costs, provided opportunities for additional genotyping, and allowed agricultural research scientists to perform a wide variety of applications, such as high-throughput genotyping by sequencing (GBS), whole-genome sequencing (WGS), genome-wide association studies (GWAS), and genomic selection. So far, most ALS resistance studies published have been conducted on biparental mapping populations with associated SNPs that often are polymorphic only in segregating populations from crosses between Andean and Mesoamerican cultivars. Genome-wide association studies will allow for finding resistance loci in a more diverse genetic background than biparental mapping, where allelic diversity is limited.

Genome-wide association studies have been used to explore the genetic basis of disease resistance, to identify new genomic regions controlling resistance, and to find molecular markers associated with ALS resistance in common bean (Perseguini et al., 2016; Zuiderveen et al., 2016; Tock et al., 2017). Perseguini et al. (2016) using GWAS detected 17 and 11 significant marker–trait associations, on a 0.05 significance level, for ALS and anthracnose resistance loci, respectively. Significantly, associated markers were distributed on most chromosomes of the genome. These authors reported that their results indicated a quantitative and complex inheritance pattern for the ALS and anthracnose diseases of common bean. Conversely, Nay et al. (2018) conducted GWAS in a large common bean panel and tested it with a mixture of five races of *P. griseola* in the field in Darien, Colombia, and with one race (63-47) in the greenhouse. A single ALS resistance locus on chromosome Pv08 was conferring resistance in the field and greenhouse trials. The GWAS results in this study suggested a qualitative nature of the ALS resistance, and a SNP that clearly distinguished resistant from susceptible bean lines was reported (Nay et al., 2018). More GWAS studies are underway to evaluate the pathotype-specific effect of resistance against prevalent races of *P. griseola* in Colombia and Uganda (M.M. Nay, unpublished data, 2018). These studies will give insights into the effectiveness of ALS resistance loci on different continents. High-density marker data obtained using GBS or WGS will enable the selection of markers that specifically tag ALS resistance loci, unlike previously developed markers that were effective in two-parental study populations but have been ineffective when used in breeding populations.

## BREEDING OF ANGULAR LEAF SPOT-RESISTANT CULTIVARS

Conventional ALS resistance breeding is based on the selection of resistant lines under field conditions using natural or artificial inoculation. Characterization of ALS resistance loci has advanced in recent years, and molecular markers linked to resistance alleles in some of the most resistant donor genotypes are available. Moreover, MAS allows for the selection of resistant lines from segregating populations using genetic analysis instead of phenotypic screening (reviewed in Miklas et al., 2006). Conversely, DNA-based selection techniques, such as marker-assisted and genomic selection, have not been routinely applied in common bean breeding, which still depends heavily on traditional techniques. In the next sections, we review some of the ALS resistance breeding strategies implemented in different breeding programs in the tropics, where ALS is a recurrent and severe disease.

## BREEDING FOR ANGULAR LEAF SPOT RESISTANCE AT CIAT HEADQUARTERS IN CALI, COLOMBIA

Breeding for resistance to ALS has long been a major objective of the bean program at CIAT in Cali, Colombia. Large collections of 22,832 wild and cultivated common beans have been screened in the field using local pathogen isolates, and highly resistant sources such as G10909, G10474, and G5686 have been identified (Pastor-Corrales et al., 1998; Mahuku et al., 2003). Pre-breeding lines of the Mesoamerican small red-seeded grain class were created by crosses and backcrosses with identified resistance sources, and ALS resistance was introgressed into elite breeding germplasm by subsequent crossing and selection under field conditions.

In the Andean breeding program, resistance sources AND 277 and CAL 143 of the Andean gene pool were used for introgressing ALS resistance. Recently, transfer of the ALS resistance of Mesoamerican origin into Andean bush types was attempted through selection by crossing Andean elite lines with ALS-resistant Mesoamerican pre-breeding lines (Solarte, 2014). A new, highly specific marker (ALS_Chr08_CT_57798588) based on the whole genome sequence of the Mesoamerican bean G10474 is now available, and it has been validated to tag *Phg2* specifically in G10474 and another Mesoamerican resistant accession G4691, but not in Mesoamerican breeding lines (Lobaton et al., 2018). Another project at CIAT is aimed at pyramiding five QTLs from the Andean (AND 277 and G5686) and Mesoamerican (G10474 and G10909) ALS resistance sources and to combine them with the well-accepted grain quality of CAL143 and KAT B1 cultivars. Single nucleotide polymorphism markers that tag ALS resistance genes have also been established at a commercial genotyping service, allowing for high-throughput screening of the progeny of crosses.

Taken together, efforts to generate ALS-resistant breeding lines at CIAT, Colombia, are ongoing due to the persistent need in Africa and other regions. Better molecular markers that are more specific, easier to use on a large scale, and tag different ALS resistance loci are becoming available and should increase the efficiency of ALS resistance breeding. Despite the availability of molecular tools, phenotypic evaluations under field conditions will remain an important method and the final quality check for selecting new varieties.

## BREEDING FOR ANGULAR LEAF SPOT RESISTANCE IN BRAZIL: RECURRENT SELECTION STRATEGY

With the expansion of irrigated areas in Brazil, common bean has become a highly valuable crop that can be grown all year. Brazil is the largest producer and the largest consumer of common bean in the world (Beebe, 2012). The increase in production has also resulted in an increase in the incidence of common bean diseases, with ALS causing significant crop damage (Singh and Schwartz, 2010; de Paula Júnior et al., 2015). As the *P. griseola* races occurring in Brazil are some of the most aggressive, it is difficult to obtain common bean lines with durable resistance (Nietsche et al., 2001; Sartorato and Alzate-Marin, 2004; Silva et al., 2008; Balbi et al., 2009; Pereira et al., 2013; Sanglard et al., 2013).

One of the methods used for breeding durable ALS-resistant cultivars in Brazil is recurrent selection (Ramalho et al., 2012). In 1998, a phenotypic recurrent selection program was initiated in the state of Minas Gerais with the goal of obtaining elite lines that accumulate important ALS resistance alleles combined with high seed yield and the carioca grain type, the market class preferred by Brazilian consumers (Amaro et al., 2007; Arantes et al., 2010). The breeding program involved a circulating diallel cross of seven carioca-seeded lines and 10 sources of ALS resistance from different market classes including Andean and Mesoamerican common bean cultivars (AN 512561, AND 277, Ouro Negro, Compuesto Negro Chimaltenango, CAL 143, MAR 2, MAR 1, G5686, MA 4.137, and Jalo). In 2011, the progress of the breeding program was evaluated, and the five best lines from Cycles I to VII were evaluated in three locations in Minas Gerais (T.I.P.O. Souza, unpublished data, 2018). The mean ALS severity scores of the lines from each recurrent selection cycle ranged from 6.2 in Cycle I in Lambari to 3.2 in Cycle III in Patos de Minas. In addition, the interactions of lines ´ locations were significant for ALS severity, which suggested that the prevalent races of *P. griseola* differ among locations and/or that environmental conditions favor the development of the disease differently among locations. On average, in the three environments, the genetic progress for ALS severity estimated by the cycle of selection was −2.9% and for seed yield was 1.8%, confirming the efficiency of a recurrent selection program. The chosen strategy, therefore, appears to have been successful in accumulating alleles for ALS resistance and for high seed yield.

Furthermore, the 27 highest performing lines from different cycles were tested in different years in the final field trials for agronomic performance evaluation of elite common bean lines conducted by Universidade Federal de Lavras–EMBRAPA in Minas Gerais. The lines MAIV 18-529 and MAIV 18-524 were selected as the most promising, and they have been used as parents (ALS elite resistance sources) in elite crosses in different breeding programs in Brazil.

## BREEDING FOR ANGULAR LEAF SPOT RESISTANCE IN EAST AFRICA

Angular leaf spot is one of the most important diseases of common bean in East Africa (Pastor-Corrales et al., 1998), with annual yield losses in Africa up to 384,200 metric tons, as estimated by Wortmann et al. (1998). In fact, this disease has been associated with up to 50% yield losses among released bean varieties in Uganda (Pastor-Corrales et al., 1998; Opio et al., 2001; Namayanja et al., 2006). Moreover, progress in breeding for ALS resistance has been slow, mainly because of the high diversity of ALS pathogen races found in East Africa (Wagara et al., 2011; Ddamulira et al., 2014b; Leitich, 2016; Kijana et al., 2017). Research on ALS in East Africa has focused on the identification and genetic characterization of new sources of resistance in local landraces and released varieties (Ng’ayu-Wanjau, 2013; Ddamulira et al., 2014a; Leitich, 2016; Kijana et al., 2017). In these studies, Mexico 54 was found to be resistant to most races of *P. griseola*, and this cultivar is the most common source of ALS resistance in breeding in Uganda and East Africa (Namayanja et al., 2006; Chilagane et al., 2013; Ng’ayu-Wanjau, 2013; Ddamulira et al., 2015; Miller et al., 2018).

Marker-assisted selection can improve breeding efficiency by facilitating introgression of resistance loci into elite cultivars, and it allows pyramiding of resistance loci for more durable resistance. In the 2000 to 2010 period, the National Agricultural Research System (NARS) common bean breeding programs in East and Central Africa had little access to MAS facilities and hence collaborated with CIAT, which had established a simple but fully functional MAS laboratory at the National Agricultural Research Laboratories (NARKL) at Kawanda, Uganda. This laboratory was and is still used for MAS projects by CIAT in collaboration with NARS for targeting disease resistance, including resistance to ALS. For example, the SCAR marker OPE4_709_, developed by Mahuku et al. (2011), and which tags *Phg-2* from Mexico 54, was used to select resistant progeny containing *Phg-2*. Using this marker, the prevalence of the *Phg-2* locus in advanced lines was assessed, and it was found that 60% of lines from Rwanda and 13% of lines from Uganda harbor the resistance allele. The lines from Rwanda had previously undergone selection at an ALS hotspot in Rubona, which explains the relatively high frequency of lines carrying the *Phg-2* resistance locus. Given the high diversity of *P. griseola* races found in Africa (Wagara et al., 2011; Ddamulira et al., 2014b; Leitich, 2016; Kijana et al., 2017), more than one resistance gene will be required to confer durable ALS resistance to a wide range of races. Ddamulira et al. (2015) pyramided resistance genes from AND 277 (*Phg-1*), Mexico 54 (*Phg-2*), and G5686 (*Phg-4*, *Phg-5*, and *Phg_G5686C_*) and introgressed the resistance into the susceptible cultivars Kanyebwa (local landrace) and CAL 96. The presence of the resistance loci was verified by molecular markers, and the resulting population showed reduced ALS symptom severity compared with single crosses when inoculated with race 61-63 (Ddamulira et al., 2015). Supported by MAS, breeding at CIAT Uganda focuses on combining ALS resistance with resistance to other stresses, and in one study, six genes for resistance to anthracnose, *Pythium*, viruses, and ALS were pyramided into four genotypes (Okii et al., 2017).

Angular leaf spot studies at CIAT Uganda have heavily relied on student projects, but the obtained knowledge about genes, markers, and new resistant germplasm is used in systematic breeding, supporting phenotypic selection in germplasm development. However, reliance on Mexico 54 as the major source of resistance has resulted in less-than-adequate progress in the desired market classes. This might be caused by the undesired characteristics introduced through crossing the small-seeded Mesoamerican cultivar Mexico 54 with large-seeded Andean beans, which are the preferred grain type in East Africa. Identification of additional effective ALS resistance sources from the Andean gene pool for use in breeding for resistance in East Africa will reduce the linkage drag and accelerate breeding progress.

## OUTLOOK ON BREEDING FOR RESISTANCE TO ANGULAR LEAF SPOT

Breeding common bean cultivars with durable resistance to *P. griseola* will continue to be a high priority in many countries of Latin America and Africa, where ALS is a recurring disease. However, durable resistance is difficult to achieve due to the extensive virulence diversity of the ALS pathogen and its capacity to produce new virulent strains. In this review, we examined the components of a durable strategy for resistance to ALS, which includes a review of the virulence diversity of the ALS pathogen, the existing situation of ALS resistance loci, and molecular markers linked to these loci for use in resistance breeding. We also consider how new technologies could facilitate and accelerate ALS resistance breeding but also disease resistance breeding in common bean in general. Different populations of the ALS pathogen have co-evolved separately with Andean and Mesoamerican common beans, such that there are distinct Andean and Mesoamerican races of the ALS pathogen. A similar pattern was observed in the common bean diseases anthracnose and rust. In rust, combining Andean and Mesoamerican resistance genes in single cultivars has resulted in resistance to all known races of the rust pathogen (Pastor-Corrales et al., 2007).

These results have important implications for the development and deployment of ALS-resistant varieties. Even though there are three Andean and two Mesoamerican ALS resistance loci, there are no common bean cultivars grown by farmers that combine Andean and Mesoamerican resistance loci that would allow evaluating whether these combinations of genes confer broad and durable resistance. The extensive virulence diversity of the ALS pathogen suggests that common bean cultivars with single genes for resistance to ALS will likely succumb to new virulent races of the ALS pathogen in the future. This has frequently been observed in bean cultivars harboring single genes for resistance to the rust and anthracnose pathogens (Kelly et al., 1994; del Río et al., 2003; Pastor-Corrales et al., 2010). In addition, there is ample evidence that the virulence diversity of the ALS pathogen is changing. New races that overcome the resistance of all differential cultivars have been found in Brazil, Central America, and, more recently, in Africa. This situation calls for a breeding strategy based on a broad diversity of quantitative and qualitative resistance, which may confer broad-spectrum and durable resistance (St. Clair, 2010). Breeding, however, is currently based on a few well-characterized resistance loci: the Mesoamerican Mexico 54 with *Phg-2* in Africa, the Mesoamerican Ouro Negro with *Phg-3* in Brazil, the Andean G5686 with *Phg-4*, and the Mesoamerican G10474 likely with *Phg-2* in Colombia. These single resistance genes are easy to transfer to new commercial cultivars, but they are also at risk of losing their resistance to new virulence races of the ALS pathogen. Thus, there is a need to discover new Andean and Mesoamerican ALS resistance genes to broaden the genetic base of common bean against the highly virulent ALS pathogen. There is also the need to learn about the spectrum of resistance of each of the ALS resistance genes by challenging them with a broad diversity of Andean and Mesoamerican races of the pathogen, and to combine (pyramid) various effective genes into commercial cultivars. Releasing and disseminating new superior varieties will stabilize bean productivity in vulnerable populations and will positively affect the livelihoods of the producers that depend on this crop for nutrition and income.
